# Vestibular Evaluation Using Videonystagmography of Chronic Zinc Deficient Patients Due to Short Bowell Syndrome

**DOI:** 10.1016/S1808-8694(15)30792-8

**Published:** 2015-10-19

**Authors:** Gustavo Duarte Paiva Ferreira, Maria Cristina Lancia Cury, José Antônio de Oliveira, Alessandra Kerli Manfredi, Hélio Vannucchi

**Affiliations:** 1 Master's degree, assistant physician of the Núcleo de Otorrinolaringologia e Cirurgia de Cabeça e Pescoço de São Paulo; 2 Habilitation degree, assistant professor of the Departamento de Otorrinolaringologia, Oftalmologia e Cirurgia de Cabeça e Pescoço, Faculdade de Medicina de Ribeirão Preto - USP; 3 Habilitation degree, full professor of the Departamento de Otorrinolaringologia, Oftalmologia e Cirurgia de Cabeça e Pescoço, Faculdade de Medicina de Ribeirão Preto - USP; 4 Doctoral degree, speech therapist of the Setor de Fonoaudiologia, Hospital das Clínicas da Faculdade de Medicina de Ribeirão Preto - USP; 5 Habilitation degree, Head of the Unidade Metabólica, Faculdade de Medicina de Ribeirão Preto - USP. Faculdade de Medicina de Ribeirão Preto da Universidade de São Paulo

**Keywords:** bowel, labyrinth, nystagmus, dizziness, thrombosis, zinc

## Abstract

The presence of zinc in the auditory pathways and its probable participation in tinnitus and hearing loss are known facts, although there are no clinical trials and experimental studies showing the impact of hypozincemia in the vestibular system and zinc existence in the vestibular pathway, respectively.

**Aim:**

This study is an attempt to correlate hypozincemia and abnormal vestibular function.

**Methods:**

This is a clinical retrospective case study where nine patients suffering of chronic zinc deficiency had their serum zinc determined and were submitted to videonystagmography. Results were compared to a normal (control) group.

**Results:**

All vestibular test parameters were altered when we compared experimental and control groups.

**Conclusion:**

Comparison between groups shows significant differences in many aspects of the vestibular analysis and calls our attention towards a possible participation of zinc on the genesis of vestibular disorders.

## INTRODUCTION

The physiological role of the zinc ion in the central nervous system (CNS) is not fully understood. Since the neural pathways in which zinc was found are glutamatergic, and knowing that zinc inhibits the binding of glutamate to its receptors, it is thought that this ion modulates the glutamatergic synapses.^1^

In 1981, zinc was found in the cochlear nuclei.

Many authors have correlated zinc physiology with the onset of tinnitus.^3^, ^4^, ^5^ Systemic administration of zinc has been suggested as an alternative therapy for treating this condition.^6^, ^7^, ^8^, ^9^ Zinc also has a role in the structure of carbonic anhydrase,^10^ which removes free radicals in the vascular stria of the cochlea. Hypozincemia may alter the effect of this enzyme on carbon dioxide metabolism in the cochlea.

The participation of zinc in calcium channels and the sodium-potassium pump was described in 1983.^11^ This pump is controlled by Na-K-ATPase and inhibited by zinc. Consequently, zinc deficiency may change the endocochlear potential, altering cochlear electrophysiology and generating tinnitus.

Changes in zinc concentration may affect the structure and function of hair cells.^12^ These authors have shown that giving ototoxic doses of gentamicin causes decreased hearing, a significant increase in perilymphatic zinc content, and decreased serum levels of zinc.

Zinc-rich diets are effective in preventing ototoxicity due to cadmium, which is the most toxic of heavy metals.^13^

There is slower conduction of electrical signals along brainstem auditory pathways in zinc-deficient patients with the short bowel syndrome.^14^

Adequate zinc supplementation in patients complaining of tinnitus decreased dizziness in all of the patients with both symptoms.^15^ According to this author, zinc appeared to affect the physiology of the posterior labyrinth by an unknown mechanism, and thus to reduce dizziness.

## OBJECTIVE

The purpose of this study was to establish whether there was any relation between non-isolated chronic zinc deficiency and altered vestibular system function.

## MATERIAL AND METHOD

The experimental group consisted of 9 subjects with the malabsorption syndrome due to the short bowel syndrome after intestinal resection following mesenteric thrombosis. These patients are monitored at a Unidade Metabólica. The control group comprised 7 subjects with no auditory or vestibular complaints.^16^

Subject in the experimental group were undernourished due to the intestinal resection; periodically they were given oligoelements by the parenteral route as inpatients at the Unidade Metabólica.

Exclusion criteria for both groups were: hearing loss due to prior exposure to noise, uncontrolled diabetes mellitus and arterial hypertension, renal failure, hormone/endocrinological disorders (including thyroid disorders), elevated triglycerides and cholesterol, chronic otitis media, neurological disorders (including oculomotor conditions), a history of neurological disease, use of drugs that act on the CNS and vestibular system, and age below 18 years.

A detailed clinical history was taken of all subjects, followed by an otoneurological evaluation consisting of a physical examination, voice and pure tone audiometry, impedance testing and videonystagmography.

Serum zinc and magnesium levels were measured before supplementation. The results of the experimental group were compared with normal reference values for those elements.^17^

Voice and pure tone audiometry, immittance testing, and videonystagmography were done on the day that patients were admitted into hospital for parenteral nutritional supplementation, before starting therapy; thus, the assessment was done when zinc levels were lowest. Videonystagmographic parameters in the experimental group were compared with control group data.

The VNG ULMER (Synapsys S.A., Marseille, France) software was used in videonystagmography. Recordings were stored in a microcomputer.

Student's “t” test was applied for comparing the data among the control and experimental groups and normal reference values. The SigmaStat 3.2 (Jandel Co.) software was used for this purpose.

The Research Ethics Committee approved this study (number 3136/2003).

## RESULTS

Age ranged from 33 to 65 years (mean 51.4 years) in the experimental group. There were three females (33%) and 6 males (67%).

Age ranged from 51 to 64 years (mean 59 years) in the control group. There were five females (71%) and 2 males (29%).

The age difference between both groups was not statistically significant (P=0.111).

The interval between zinc supplementation ranged from 2 to 30 days.

All 9 subjects used medication regularly. Five subjects (55%) used anticoagulants, 2 (22%) used vasodilators, and 4 used antihypertensive drugs (44%).

Ototoxic drugs were used preoperatively by 100% of subjects.

All subjects had a history of mesenteric thrombosis, treated by intestinal resection. Eight subjects had comorbidities; systemic arterial hypertension was the most frequent (4 cases – 44%). Two subjects had chagasic myocardiopathy (22%). Other conditions were thromboangiitis obliterans, bilateral glaucoma, and non-dialysis-dependent chronic renal failure, one case each (11%).

The otoneurological exam revealed tympanic perforation in one subject (11%), a positive Romberg-Barré in 3 subjects (33%), segment deviation in 1 subject (11%), positional semispontaneous nystagmus in 1 subject (11%), gait deviation in 2 subjects (22%), and a widened walking base in 1 subject (11%).

Four subjects reported dizziness in caloric testing (44%).

Serum zinc and magnesium values were compared to reference values. This comparison showed statistical significance for both zinc and magnesium.

Calibration was regular in all tests.

There was a statistically significant difference in the calibration value, the velocity to the left, and right and left precision when comparing both groups.

Spontaneous nystagmus was absent in all tests in both groups.

No subject in both groups presented semispontaneous nystagmus.

A comparison between both groups revealed a statistically significant difference in left horizontal saccadic movement latency, right and left precision, and upward vertical saccadic latency.

Four (44.4%) of 9 subjects in the experimental group had type I tracking, 3 subject (33.3%) had type II tracking, and 2 subject (22.2%) had type III tracking.

The qualitative analysis of pendular tracking was not done in the control group.

A comparison between both groups showed that there was a statistically significant difference in right and left gain.

All of the horizontal responses were symmetrical. One subjects had asymmetrical vertical responses.

A comparison between both groups showed that there was a statistically significant difference in velocity and gain for the horizontal optokinetic nystagmus.

All patients except for one subject that presented bilateral chronic otitis media performed caloric testing. The air caloric test was not applied to subject to maintain uniformity in the method.

On subject presented bilateral hyporeflexia.

Ocular fixation with the inhibitory stimulus was attained in all tests. No inversion of nystagmus was recorded in any test.

Slow component acceleration velocity values upon warm stimulation of the right ear ranged from 3.5 to 60 degrees/second (g/s); the mean value was 21.02 g/s and the standard deviation was 20.56. The slow component acceleration velocity upon cold stimulation of the right ear ranged from 5.3 to 47.1 g/s; the mean value was 19.08 g/s and the standard deviation was 15.29. The slow component acceleration velocity upon warm stimulation of the left ear ranged from 4.8 to 79.6 g/s; the mean value was 20.67 g/s and the standard deviation was 27.63. Cold stimulation yielded slow component acceleration velocity values ranging from 3.8 g/s to 35.8 g/s; the mean value was 17.9 g/s and the standard deviation was 10.38.

A comparison of reflectivity and directional preponderance between the two groups did not yield statistically significant values.

Only one subject in the control group had a deficit to the right; thus, statistical analysis tests and a comparison between groups was not possible.

There was no statistically significant difference between groups in deficits to the left.

## DISCUSSION

Although a variety of substances are absorbed along the small intestine, each of them has a main absorption site. When parts of the intestine are injured or resected, generally the remaining portions adapt to effectively absorb the substances that, under normal conditions, would have been absorbed by the injured or resected portions. There are two noteworthy exceptions to this adaptation process: cobalamine (vitamin B12) and biliary salts. An individual from whom the distal portion of the ileum is resected will never again absorb these substances actively.^18^

Aminoglycoside antibiotic use and postoperative observation in intensive care unit are common measures in subjects undergoing intestinal resection. All subjects in our sample were given ototoxic drugs during and after intestinal resection surgery; according to our findings, this did not affect vestibular function, as we will see below.

Magnesium was measured with zinc, since their absorption and serum elevation/reduction behavior is similar. However, magnesium was not the focus of this study.

There was a significant difference between zinc and magnesium serum levels in the experimental group and the mean normal reference range for these elements, which reinforces the idea of malabsorption syndrome or disabsortive disease in the experimental group.

Five subjects had bilateral sensorineural dysacusis, as evidenced in audiometric testing. One subject had bilateral mixed dysacusis, one subjects had unilateral conductive dysacusis, and one exam was within normal limits. Audiometry was not done in one subject. A predominance of sensorineural hearing loss was expected due to the mean age of the sample, a high prevalence of cardiovascular comorbidities, and use of ototoxic drugs.

Vestibular function was investigated using videonystagmography, since this method is more sensitive and precise than others, and makes it possible to eliminate artifacts and to record ocular movements in all directions.^16^^,^^19^, ^20^

Although calibration was regular in all cases, all the parameters (values, latencies, velocities and precision) were altered; there were significant differences in value, velocity to the left and precision to the right and left, all of which clearly demonstrate poor functioning of specific CNS neuronal pathways, although the cerebellum appeared to be normal. In cerebellar syndromes, tracings are characteristically irregular and anarchic.

Saccade test evaluate the efficiency of CNS control of rapid eye movements.

Saccades in the experimental group were significantly altered, as seen in horizontal latencies to the left, upward vertical latencies, and right and left precision. It should be mentioned that saccades were tested with frequency stimuli at one tenth of the calibration frequency (0.1 Hz), and were likewise significantly altered, which showed that neural connections responsible for the CNS vision-vestibular system interaction were compromised. Topographically, this may occur from the cortex to the extraocular muscles, passing through the brainstem, the cerebellum, and the vestibular and oculomotor nuclei.

Both groups differed significantly in terms of right and left pendular gain. The mean gain for both sides was lower than the minimum normal reference value.

Any point of the efferent pathway, from the cortex to the oculomotor muscles and extraocular muscles could be affected; this included the pons, the midbrain, the cerebellum and the vestibular nuclei, all of which are intermediate stations in that pathway.

Low gain in pendular tracking may be due to medication, disorders of vision, lack of attention in the examinee, and CNS conditions such as diffuse cerebellar, brainstem and basal ganglia lesions. These conditions may alter tracking bilaterally. Unilateral (ipsilateral) changes may be seen in brainstem and cerebellopontine angle injuries.^19^

Findings in the experimental group, compared with the control group, suggest once again that there is central involvement in these subjects, especially in the oculomotor and tracking systems. Tests showed symmetrical or coordinated tracking reflexes that were not proportional (low gain) to the path taken by the object (target).

Response asymmetry was seen in a vertical optokinetic test, as well as significant gain and velocity differences in the horizontal optokinetic test; this underlines the findings associated with tracking and suggests central involvement.

The Otoneurology Unit uses water in the caloric test; they believe this method is more reliable than using air in this test. Thus, the caloric test was not done in the subject that had tympanic perforation, to avoid departing from the chosen method.

A comparison of reflectivity and directional preponderance between groups did not reveal significant differences. All subjects in the experimental group, except for one subject, responded to the caloric test as subjects in the control group.

We deduced that the function of the posterior labyrinth, specifically the sensory hair cells in the lateral semicircular canals, was normal in subjects belonging to the experimental group. Thus, ototoxic drugs could not be considered responsible for the changes found in other tests.

Vestibular system functioning is due to a complex system that integrates multisensory information involving many well-defined reflexes;21 our findings thus revealed that the connection between the CNS and the labyrinth and ocular apparatus was compromised. These were changes suggesting poor functioning of one or more specific neuronal pathways, possibly the vestibulo-oculomotor pathway, since there were interactions between vestibular and oculomotor nuclei at the brainstem.

Such involvement may occur in the cerebellum, since saccades, tracking and optokinetic tests showed changes suggesting involvement of the cerebellum and/or vestibular nuclei. These affect the vestibulo-ocular and vestibulo-cerebellar reflexes, acting as an important sensorimotor integration center. Other ascending pathways may also be involved, such as the vestibulo-cerebellar and vestibulo-cortical pathways and the medial longitudinal fascicle, essential for adequate vestibulo-ocular reflex function.^22^

Glutamate and GABA are two of the neurotransmitters in afferent and efferent vestibular endings and in synapses between the vestibular nerve and the vestibular nuclei.^22^ Zinc has been systematically found in glutamatergic synapses,^23^ which consequently have been named gluzinergic synapses. Zinc also has a modulating role in glutamatergic and GABAergic synapse neurotransmission.^24^^–26^ Thus, it is reasonable to assume that zinc may also be present in the vestibular pathways, acting in oculomotor response control functions. There are few published papers on this topic in the literature.

Detection of central vestibular changes in this sample of patients with the malabsorption syndrome due to extensive intestinal resection has led us to believe that zinc deficiency, among other nutrients that need to be replenished, may affect the function of the aforementioned pathways. Additional studies are needed to investigate the issue further.

## CONCLUSION

A comparison between the experimental and control groups revealed significant differences in various parameters of the vestibular evaluation, and brings attention to a possible role of disabsortive alterations in the origin of vestibular lesions.
